# Editorial: MicroRNA Signatures in Plant Genome Stability and Genotoxic Stress

**DOI:** 10.3389/fpls.2021.683302

**Published:** 2021-04-22

**Authors:** Anca Macovei, Ignacio Rubio-Somoza, Jorge Almiro Pinto Paiva, Susana Araújo, Mattia Donà

**Affiliations:** ^1^Department of Biology and Biotechnology “Lazzaro Spallanzani”, University of Pavia, Pavia, Italy; ^2^Molecular Reprogramming and Evolution Laboratory (MoRE), Centre for Research in Agricultural Genomics (CRAG), Barcelona, Spain; ^3^Institute of Plant Genetics of the Polish Academy of Sciences, Poznan, Poland; ^4^Instituto de Biologia Experimental e Tecnológica, Oeiras, Portugal; ^5^Association BLC3, Technology and Innovation Campus, Centre BIO–R&D Unit, Lagares da Beira, Portugal; ^6^Instituto de Tecnologia Química e Biológica António Xavier, Universidade Nova de Lisboa, Oeiras, Portugal; ^7^Gregor Mendel Institute of Molecular Plant Biology, Austrian Academy of Sciences, Vienna BioCenter (VBC), Vienna, Austria

**Keywords:** MicroRNAs, genotoxicity, DNA damage response, DNA repair, genome stability

Plants are subjected to high levels of DNA damage resulting from their essential dependence on sunlight and exposure to environmental stresses. Accumulation of DNA lesions generated by genotoxic stress disturb genome stability, which hinders plant development and crop productivity. To ensure the maintenance of genome stability, plants developed a range of mechanisms aiming at detecting and repairing DNA damage. The intricated DNA damage response (DDR) network consists of an impressive array of DNA damage sensing and signal transduction pathways leading to DNA repair and cell survival or, alternatively, triggering cell death. Although DDR is highly conserved in eukaryotes, peculiar plant-specific features are described (Yoshiyama et al., 2013; Nikitaki et al., 2018; Nisa et al., 2019). DDR is generally far less studied in plants as compared to mammals (Gimenez and Manzano-Agugliaro, 2017). Due to the essential role of DNA repair in maintaining genomic stability, tightly controlled regulatory mechanism are required, where different players, including phytohormones (Donà et al., 2013) or epigenetic regulators (Kim, 2019), are implicated.

The role played by miRNAs on the post-transcriptional regulation of the DDR has been less considered, particularly in plants when compared to animal systems. Indeed, miRNAs-mediated regulation of DDR gene expression has been already demonstrated in mammalian systems, highly explored in view of therapeutic applications (He et al., 2016; Majidinia and Yousefi, 2016; Rezaeian et al., 2020). In plants, the role of miRNAs in the regulation of DNA damage sensing and repair mechanisms remains to be elucidated.

The present collection of articles gathered within the scope of this Research Topic aims to focus on the implication of miRNAs in genome integrity and response to genotoxic stresses, through direct or indirect interactions with DDR components.

In an extensive review article, Cimini et al. discussed the interconnections between DDR and redox systems, painting a dynamic picture intertwined with regulatory mechanism mediated by miRNAs. Based on their literature analysis, the authors propose a triangular model for redox balance, DDR and miRNAs, where reactive oxygen species (ROS) act as common denominators. At the level of the nucleus, accumulation of ROS results in excessive DNA damage and cell cycle inhibition whereas DDR aid plants to cope with these alterations. Disturbances in the antioxidant and oxidant balance can influence cell cycle progression. Hence, ROS and redox signals are involved in the regulation of gene expression at transcriptional (redox-sensitive transcription factors) and post-transcriptional (miRNAs) levels. The manuscript enlists a series of miRNAs targeting genes involved in ROS production and scavenging along with putative miRNAs implicated in DDR-associated pathways. While more miRNAs associated with ROS metabolism have been experimentally tested, in the case of DDR most examples arrive from *in silico* studies.

Similarly, the review work by Chowdhury and Basak reinforce the fact that only few plant miRNAs have been identified as active players in combating genotoxic stresses and underlines specific challenges related to miRNA research in this context. As previously mentioned, the lack of in-depth information is a consequence of DDR being significantly less studied in plants compared to animals (Hurdle I) and this combines with the limited information on miRNA targets specifically involved in coping with genotoxic stress (Hurdle II). To address these hurdles, the authors encourage the development of more focused experimental designs combined with the application of omics techniques.

Differently, the review work by Valdés-López et al. provides a different vision on the topic, by looking into the Argonaute (AGO) proteins and their implications in the symbiotic relations between bacteria and legumes, with a stopover on DNA damage/repair-related issues. Being a highly complex process, the regulation of legume-rhizobia symbiosis (LRS) is genetically controlled while some miRNAs are known to play active roles in the post-transcriptional regulation of LRS. AGO proteins (key players in all small-RNA-guided gene-silencing processes) also appear to be involved in LRS, supported by evidence that legumes possess more genes coding for these proteins compared to other non-symbiotic species. In relation to DDR, it is underlined that during rhizobial infection, nodule cells are subjected to endoreduplication, a plant-specific response to DNA damage, cell-cycle arrest, and cell death. Weather AGO proteins with known roles in DNA repair (AGO2, AGO9) are involved in this specific process, still remains to be elucidated.

The “xenomiR” hypothesis proposes that miRNAs can be transferred from one species to another and potentially target genes across distant species. The research work by Bellato et al. combine this cross-kingdom topic with the evolutionary conserved DDR pathway aiming to answer if plant miRNAs could target DDR-related processes in both plant and human cells. The authors have developed a series of bioinformatic approaches to investigate and compare miRNA targets, attesting that these methodologies can be standardized for different species whereas the generated results can serve as starting point for experimental validation of such data. This work succeeded to identify a list of miRNAs predicted to target genes involved in DNA repair, recombination, and replication, chromatin remodeling, cell cycle, and cell death in the model legume *Medicago truncatula*.

Finally, as a follow up of the bioinformatic study by Bellato et al., the research work of Gualtieri et al. provides experimental evidence on the involvement of selected miRNAs in DDR-associated pathways. The authors have developed an experimental system based on the use of specific chemical agents (camptothecin and NSC120686) know to inhibit the activity of topoisomerase 1 and tyrosyl-DNA phosphodiesterase 1 enzymes. These chemical agents were found to affect the DDR as evidenced by the accumulation of DNA damage and cell death combined with altered transcription profiles of key DDR players in DNA repair and cell cycle regulation. The expression of miRNA-target gene pairs was investigated evidencing that when a miRNA is upregulated, its predicted DDR-target gene is downregulated. The contrasting expression profiles observed support the evidence that these miRNAs (miR156a, miR172c-5p, miR2600e, miR395e, and miR5741a) could repress the expression of these targets (*UBE2A, RAD54-like, 5AT, DMAP1*, and *E2FE-like*).

Overall, this collection of articles attentively underlines the gaps-of-knowledge existing with regards to the miRNA-mediated regulation of DDR in plants, while encouraging further research still needed to shed light on this complex topic.

## Author Contributions

All authors equally contributed to write the Editorial and discuss about the articles published in the Research Topic.

## Conflict of Interest

The authors declare that the research was conducted in the absence of any commercial or financial relationships that could be construed as a potential conflict of interest.

## References

[B1] Donà M. Macovei A. Faè M. Carbonera D. Balestrazzi A. (2013). Plant hormone signaling and modulation of DNA repair under stressful conditions. Plant Cell Rep. 32, 1043–1052. 10.1007/s00299-013-1410-9 23508254

[B2] Gimenez E. Manzano-Agugliaro F. (2017). DNA damage repair system in plants: a worldwide research update. Genes 8:299. 10.3390/genes8110299 29084140 PMC5704212

[B3] He M. Zhou W. Li C. Guo M. (2016). MicroRNAs, DNA damage response, and cancer treatment. Int. J. Mol. Sci. 17:2087. 10.3390/ijms17122087 27973455 PMC5187887

[B4] Kim J.-H. (2019). Chromatin remodeling and epigenetic regulation in plant DNA damage repair. Int. J. Mol. Sci. 20:4093. 10.3390/ijms20174093 31443358 PMC6747262

[B5] Majidinia M. Yousefi B. (2016). DNA damage response regulation by microRNAs as a therapeutic target in cancer. DNA Repair 47, 1–11. 10.1016/j.dnarep.2016.09.003 27697364

[B6] Nikitaki Z. Holá M. Donà M. Pavlopoulou A. Michalopoulos I. Angelis K. J.. (2018). Integrating plant and animal biology for the search of novel DNA damage biomarkers. Mutat. Res. 775, 21–38. 10.1016/j.mrrev.2018.01.001 29555027

[B7] Nisa M. U. Huang Y. Benhamed M. Raynaud C. (2019). The plant DNA damage response: signaling pathways leading to growth inhibition and putative role in response to stress conditions. Front. Plant Sci. 10:653. 10.3389/fpls.2019.00653 31164899 PMC6534066

[B8] Rezaeian A.-H. Khanbabaei H. Calin G. A. (2020). Therapeutic potential of the miRNA-ATM axis in the management of tumor radioresistance. Cancer Res. 80, 139–150. 10.1158/0008-5472.CAN-19-1807 31767626

[B9] Yoshiyama K. O. Sakaguchi K. Kimura S. (2013). DNA damage response in plants: conserved and variable response compared to animals. Biology 2, 1338–1356. 10.3390/biology2041338 24833228 PMC4009792

